# Unified Approach to Treating Exogenous Endophthalmitis With Immediate Vitrectomy

**DOI:** 10.1177/24741264241275246

**Published:** 2024-09-11

**Authors:** Graeme K. Loh, Amit V. Mishra, Mark Seamone, Matthew Tennant

**Affiliations:** 1 Department of Ophthalmology and Visual Sciences, University of Alberta, Edmonton, AB, Canada

**Keywords:** exogenous endophthalmitis, acute endophthalmitis, early vitrectomy, immediate vitrectomy, small gauge, pars plana vitrectomy

## Abstract

**Purpose:** To analyze the outcomes and complications of immediate (within 24 hours) small-gauge (23-gauge, 25-gauge) pars plana vitrectomy (PPV) for all causes of exogenous endophthalmitis. **Methods:** A retrospective case series was evaluated. **Results:** The study included 107 patients who had immediate PPV for exogenous endophthalmitis between 2016 and 2022. The primary outcome measures were the change from baseline best-corrected visual acuity (BCVA) to the final follow-up and the complications after PPV. Causes of exogenous endophthalmitis included intravitreal injections (62.6%), PPV (18.7%), cataract surgery (11.2%), glaucoma surgery (5.6%), and trauma (1.9%). The most common complications were retinal detachment (17.8%) and secondary glaucoma (9.3%). The mean logMAR BCVA improved significantly from the initial diagnosis to the final follow-up (2.26 vs 1.21) (*P* < .0001). **Conclusions:** In most cases, immediate small-gauge vitrectomy for exogenous endophthalmitis leads to an improvement in VA, regardless of the VA at presentation. Patients should be counseled about the potential risks associated with PPV surgery.

## Introduction

Exogenous endophthalmitis after incisional eye surgery or trauma can have a devastating effect on a patient’s vision and quality of life. Timely diagnosis and management are crucial in mitigating the damage caused by the infection. The Endophthalmitis Vitrectomy Study (EVS),^1^ which guides vitreoretinal surgeons on when to consider immediate PPV in exogenous endophthalmitis, is now 28 years old and remains the only such randomized control trial.

Vitrectomy techniques and equipment have changed significantly since the EVS was performed. Smaller gauge PPV (23-gauge, 25-gauge, 27-gauge), higher vitrector cut rates, better widefield viewing systems, and surgical microscopes have enhanced the amount of vitreous removal that can be safely performed in eyes with exogenous endophthalmitis while reducing postoperative complications. Antibiotic regimens have also evolved since the EVS, with intravitreal (IVT) ceftazidime replacing IVT amikacin and systemic oral fluroquinolones, such as moxifloxacin or ciprofloxacin, being used as adjuncts.^2^

Since the EVS was published, immediate vitreous biopsy combined with IVT antibiotics, or tap and inject (T&I), has been the only “level 1” gold standard for eyes with exogenous endophthalmitis and a visual acuity (VA) better than light perception (LP). However, there is a growing body of evidence that immediate vitrectomy can result in better outcomes.^3–9^ To our knowledge, this is the largest consecutive case series of immediate vitrectomy for all-cause exogenous endophthalmitis. The vitreoretinal surgeons at our tertiary referral center believe that, when possible, immediate PPV for exogenous endophthalmitis is the best management, and they have taken a unified approach to this sight-threatening disease.

## Methods

This retrospective case series comprised consecutive patients who had immediate vitrectomy (within 24 hours of diagnosis) for all causes of exogenous endophthalmitis between January 1, 2016, and December 31, 2022. Institutional review board/ethics committee approval (Pro00134542) was obtained for this study.

A minimum follow-up of 6 months after primary PPV was required, and all follow-up visits until August 1, 2023, were included. Any case with a final diagnosis of endogenous or sterile endophthalmitis was excluded. No age limit was set. To enhance the accuracy and reliability of data collection, data were collected from the 7 years after patient records became fully electronic (in 2016). Records were searched through the Healthquest electronic medical records database (Microquest, Inc) and filtered with the search terms “endophthalmitis,” “PPV,” or a combination of terms.

Data collection included patient sex, age, date of primary PPV, procedure or event that caused the exogenous endophthalmitis, duration of symptoms until PPV, duration from diagnosis to PPV, laterality, preoperative T&I if diagnosed out-of-hours or from an external provider, follow-up duration, postoperative complications, and the results of Gram stain and of vitreous or aqueous samples. The preoperative BCVA and postoperative BCVA at 3 months, 6 months, and the final follow-up were also collected. The VA was recorded in both Snellen and logMAR notations.

All patients diagnosed with exogenous endophthalmitis out-of-hours (Monday to Sunday evenings and overnight) were given immediate T&I before vitrectomy the next day. At the time of diagnosis, patients received an immediate dose of 800 mg oral moxifloxacin, which was subsequently followed by a daily 400 mg dose for 6 days.

PPV was performed at the same institution by 8 vitreoretinal surgeons with a 23- or 25-gauge vitrectomy setup. A vitreous biopsy with a vitrector or needle aspirate was performed in all cases. Comprehensive and complete vitreous removal, including posterior vitreous detachment induction, was performed as safely as possible at the surgeon’s discretion. IVT antibiotics and steroid injections were standardized (vancomycin, 1 mg in 0.1 mL; ceftazidime, 2.25 mg in 0.1 mL; dexamethasone, 1 mg in 0.1 mL) for both PPV and T&I. Subconjunctival dexamethasone and cefazolin were administered after completion of all PPVs.

Statistical analyses were performed using SPSS software (version 28, IBM Inc). A paired *t* test was used to compare the logMAR BCVA before PPV, 3 months and 6 months postoperatively, and at the final follow-up. Statistical significance was set at *P* < .05.

## Results

This study included 107 eyes of 107 patients with a mean age of 74 years (range, 8-96). The mean duration of symptoms until PPV was performed was 2.2 days (range, 0-7). All causative events occurred within 4 weeks of a diagnosis of exogenous endophthalmitis. Table 1 shows the baseline characteristics of the patients. The mean follow-up was 25.2 months (range, 6-74).

**Table 1.** Baseline Characteristics of Patients.

Characteristic	Value
Age (y)	
Mean	
Range	74
Sex, n (%)	8, 96
Male	48 (44.9)
Female	59 (55.1)
Laterality, n (%)	
Right	54 (50.5)
Left	53 (49.5)
Duration of symptoms until PPV (d)	
Mean	2.2
Range	0, 7
Duration of diagnosis until PPV (d)	
Mean	0
Range	0, 1
Single T&I before PPV, n (%)	29 (27.1)
Presence of hypopyon at presentation, n (%)	107 (100)

Abbreviations: T&I, tap and inject; PPV, pars plana vitrectomy.

### Causative Events

Table 2 shows the causes of exogenous endophthalmitis. The most frequent cause was previous IVI followed by PPV, cataract surgery, glaucoma filtration surgery, and trauma. The occurrence of exogenous endophthalmitis after IVI was approximately 1 in 4500, which is comparable to previously reported rates; after PPV, it was approximately 1 in 600, a higher rate than expected. Nine of 20 cases of exogenous endophthalmitis after PPV (45%) occurred during insertion of an Akreos intraocular lens (IOL) (Bausch + Lomb); 5 of the cases occurred during the early adoption period of implanting this IOL. The surgical technique was modified to include suturing of all vitrectomy port sclerotomies to reduce the risk for postoperative hypotony. The rate of exogenous endophthalmitis after Akreos IOL insertion decreased significantly after this modification despite a steady increase in the number of IOLs implanted.

**Table 2.** Causative Event Leading to Exogenous Endophthalmitis.

Event	Number (%)
IVT injection	67 (62.6)
PPV	20 (18.7)
Cataract surgery	12 (11.2)
Glaucoma filtration surgery	6 (5.6)
Trauma	2 (1.9)

Abbreviations: IVT, intravitreal; PPV, pars plana vitrectomy.

### Causative Organisms

Table 3 shows the culture results of the vitreous biopsy. Of the 63 culture-positive cases (58.9%), the most commonly identified organisms were *Staphylococcus epidermidis*, followed by *Enterococcus faecalis, Streptococcus viridans*, and *Staphylococcus aureus*. No fungal isolates were identified. Forty cases (37.4%) were culture negative, and 4 cases (3.7%) were missing samples.

**Table 3.** Vitreous Biopsy Culture Results.

Result	Number (%)
Gram-positive bacteria	58 (92.2)
*Staphylococcus epidermidis*	36 (57.1)
*Enterococcus faecalis*	7 (11.1)
*Streptococcus viridans*	4 (6.3)
*Staphylococcus aureus*	4 (6.3)
*Streptococcus pneumoniae*	1 (1.6)
*Staphylococcus hominis*	1 (1.6)
*Streptococcus dysgalactiae*	1 (1.6)
*Micrococcus luteus*	1 (1.6)
*Granulicatella adiacens*	1 (1.6)
*Abiotrophica defectiva*	1 (1.6)
*Streptococcus sanguinis*	1 (1.6)
Gram-negative bacteria	5 (7.9)
*Haemophilus influenzae*	1 (1.6)
*Capnocytohaga ochracea*	1 (1.6)
*Citrobacter koseri*	1 (1.6)
*Moraxella nonliquefaciens*	1 (1.6)
*Serratia macrescens*	1 (1.6)
Culture negative	40 (37.4)
Missing samples	4 (3.7)

### Visual Acuity

**Figure 1.**
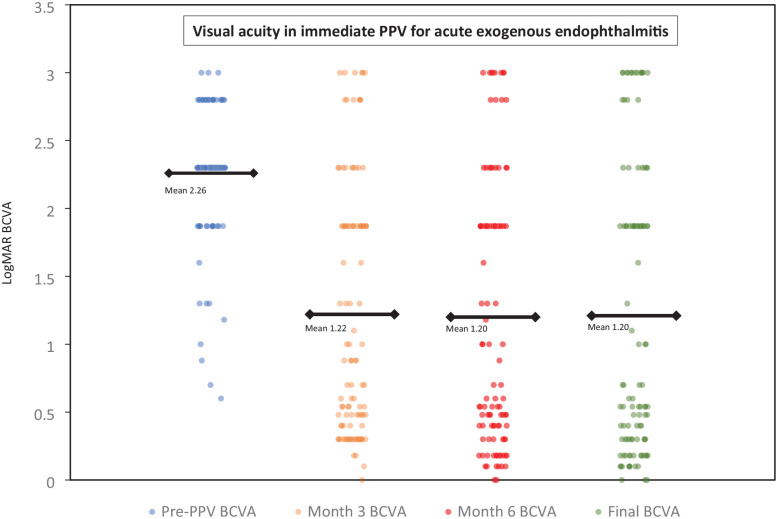
Categorical scatterplot of the logMAR BCVA before PPV and after PPV (month 3, month 6, final follow-up) for acute exogenous endophthalmitis. Abbreviations: BCVA, best-corrected visual acuity; PPV, pars plana vitrectomy.

On presentation, the VA was no LP (NLP) in 2 eyes (1.9%), LP in 22 eyes (20.6%), hand motions (HM) in 60 eyes (56.1%), and counting fingers (CF) in 14 eyes (13.1%). In 9 eyes (8.4%), the VA at presentation ranged from 20/80 to 20/800. The logMAR BCVA 3 months after PPV, 6 months after PPV, and at the final follow-up was 1.22, 1.20, and 1.21, respectively, compared with 2.26 before PPV (*P* < .0001 for all 3 timepoints). The VA improved after immediate vitrectomy in 74 eyes (89.2%), all of which had a baseline VA better than LP. Forty eyes (37.4%) had a final BCVA of 20/50 or better. The final BCVA ranged from 20/60 to 20/80 in 14 eyes (13.1%), 20/100 to 20/800 in 9 eyes (8.4%), CF in 20 eyes (18.7%), and HM in 7 eyes (6.5%). Seventeen eyes (15.9%) had LP or worse VA. The VA was NLP in 7 eyes (6.5%) at 6 months and in 13 eyes (12.1%) at the final follow-up (Figure 1).

### Postoperative Complications

Table 4 shows the postoperative complications. The most frequent complication was retinal detachment (RD) followed by secondary glaucoma and corneal decompensation. Thirty-five eyes (32.7%) required additional surgery, including repeat PPV (further anterior or posterior chamber washout with repeat IVT antibiotics, RD, silicone oil removal), scleral buckling, corneal graft placement, glaucoma filtration, and enucleation.

**Table 4.** Complications After PPV for Exogenous Endophthalmitis.

Complication	Number (%)
Retinal detachment	19 (17.8)
Secondary glaucoma	10 (9.3)
Corneal decompensation	6 (5.6)
Long-term silicone oil tamponade	3 (2.8)
Hypotony	3 (2.8)
Enucleation	2 (1.9)
IOL subluxation	2 (1.9)
Choroidal hemorrhage	1 (0.9)
Chronic cystoid macular edema	1 (0.9)
Submacular hemorrhage	1 (0.9)

Abbreviations: IOL, intraocular lens; PPV, pars plana vitrectomy.

### Retreatment

Eleven cases (10.3%) required retreatment to control the exogenous endophthalmitis. The retreatment comprised PPV with IVT antibiotics and dexamethasone or administration of IVT antibiotics and dexamethasone injections alone. Two eyes that received IVT antibiotics and dexamethasone injections alone and 9 eyes that had repeat PPV with IVT antibiotics and dexamethasone required additional IVT injections to control the exogenous endophthalmitis.

The mean duration of symptoms to the diagnosis of exogenous endophthalmitis was rapid (2.2 days), likely as a result of rigorous patient education regarding the symptoms of endophthalmitis and a 24-hour direct telephone line to an on-call retina specialist. The BCVA was better at the final follow-up than at baseline in 97 patients. Gram-negative organisms were cultured in only 2 of 20 patients who had no improvement in BCVA at the final follow-up. This can be partially explained by preexisting comorbidities, such as age-related macular degeneration (10/20 cases [50.0%]) that progressed over time. Given the relatively long follow-up, some of these patients eventually developed geographic atrophy (GA) that affected their central vision.

Twenty-nine patients (27.1%) received an out-of-hours T&I before PPV the next day. Sixty-four eyes (82.1%) not receiving a preoperative T&I and 23 eyes (79.3%) that did receive a preoperative T&I experienced visual gains. There was no statistical difference between the 2 groups. Eyes receiving an out-of-hours T&I before an in-hours PPV did not have significantly poorer visual outcomes at 6 months.

## Conclusions

There are clear advantages to performing an immediate PPV in the setting of exogenous endophthalmitis, the most significant of which is the ability to surgically clear the nidus of infection under adequate periocular anesthesia. This greatly reduces the intraocular bacterial load and has a compound effect on bacterial doubling times. Inflammatory mediators and debris that are highly toxic to the posterior pole and peripheral retina are also markedly reduced. It is debatable whether the improved access and quantity of diagnostic sampling in PPV compared with T&I truly alter management given the very high degree of susceptibility of vancomycin and ceftazidime to causative bacteria.^2^ The caveat is whether the culture determined the causative agent to be fungal, rather than bacterial, in nature.

The surgical risks of immediate PPV must be balanced against the advantages. Modern small-gauge vitrectomy mitigates the risks somewhat compared with the results in the EVS using 20-gauge instruments and non-widefield viewing systems. In our study, the complications after PPV were relatively common. Our approach to exogenous endophthalmitis also considers the challenges of after-hours operating room access by performing immediate T&I in the evenings and overnight before PPV the next day. Given the low incidence and high morbidity of exogenous endophthalmitis, there is a strong justification for immediate in-hours PPV because it has been shown to reduce retreatments compared with primary T&I.^5^

The most significant complications after immediate PPV for exogenous endophthalmitis were RD (17.8%) and secondary glaucoma (9.3%). Although these occur relatively infrequently, patients must be adequately counseled before surgery about potential and substantial ocular comorbidities. RDs after PPV in the setting of endophthalmitis are not straightforward cases and must be handled carefully.

This case series has limitations. It was not prospective or randomized and did not have a control group. Given the unified treatment approach by the surgeons in this patient population, in which immediate PPV (within 24 hours) was considered the gold standard, inclusion of a comparative group with primary T&I and delayed PPV was not possible. Some surgeons obtained undiluted vitreous samples, while others may have obtained diluted vitreous samples containing a balanced salt solution. Undiluted sampling could have increased culture-positive yields.

Many studies focus on acute exogenous endophthalmitis after cataract surgery given that it is the most common surgical procedure performed worldwide.^10^ An estimated 20 million IVT injections are given annually worldwide.^11^ The pooled incidence of exogenous endophthalmitis is 0.028% (approximately 1 in 3500).^12^ With the advent of IVT injections approved by the US Food and Drug Administration to treat GA, the number of injections administered is expected to increase. An increase in cases of exogenous endophthalmitis after IVT injections will likely be seen in our ophthalmology clinics.

In conclusion, regardless of the presenting VA, the BCVA outcomes are better when immediate PPV is performed within 24 hours of a diagnosis of acute exogenous endophthalmitis, with or without previous out-of-hours T&I. Although complications do occur, they lead to severe vision loss in only a small proportion of cases. This study adds to a growing opinion among retina specialists that immediate PPV for acute exogenous endophthalmitis is a reasonable and effective treatment modality.
